# Supplementary material for: The surface adsorption, aggregate structure and antibacterial activity of Gemini quaternary ammonium surfactants with carboxylic counterions
Source: R Soc Open Sci. 2019 Aug 28;6(8):190378. doi: 10.1098/rsos.190378 (PMC6731746; doi:10.1098/rsos.190378)
Supplement: Supplementary Material Information [file rsos190378supp1.docx]

**All the surfactants were structurally characterized by Fourier transform infrared spectroscopy and mass spectroscopy. The details of these spectral characterizations were as follows.**

11-2-11-2HCOO^−^: Mass Spectra: Cationic scanning *M/E*: 285.3; Anion scanning *M/E*: 45.0; FTIR Spectra (KBr, v, cm^−1^): 3419.38 (N-H), 3000~2800 (C-H), 1250~1100 (C-H), 1648.39 (C=O), 1593.10 (N-H), 1487.79, 1468.17 (C-H), 953.23 (C-O), 761.47 (C-H).

11-2-11-2CH_3_COO^−^: Mass Spectra: Cationic scanning *M/E*: 285.3; Anion scanning *M/E*: 59.0; FTIR Spectra (KBr, v, cm^-1^): 3293.26 (N-H), 3000~2800 (C-H), 1250~1100 (C-H), 1648.11 (C=O), 1590.39 (N-H), 1490.78, 1466.48 (C-H), 953.79 (C-O), 760.83 (C-H).

11-2-11-2CH_3_CHOHCOO^−^: Mass Spectra: Cationic scanning *M/E*: 285.3; Anion scanning *M/E*: 88.9; FTIR Spectra (KBr, v, cm^-1^): FTIR Spectra (KBr, v, cm^-1^): 3382.12 (N-H), 3000~2800 (C-H), 1300~1150 (C-H), 1651.98 (C=O), 1595.32 (N-H), 1465.62, 1375.58 (C-H), 1126.82 (O-H), 947.47 (C-O), 764.98 (C-H).

13-2-13-2HCOO^−^: Mass Spectra: Cationic scanning *M/E*: 285.3; Anion scanning *M/E*: 45.0; FTIR Spectra (KBr, v, cm^-1^): 3257.55 (N-H), 3000~2800 (C-H), 1250~1100 (C-H), 1664.15 (C=O), 1598.57 (N-H), 1487.87, 1466.65 (C-H), 949.64 (C-O), 763.71 (C-H).

13-2-13-2CH_3_COO^−^: Mass Spectra: Cationic scanning *M/E*: 285.3; Anion scanning *M/E*: 59.0; FTIR Spectra (KBr, v, cm^-1^): 3257.55 (N-H), 3000~2800 (C-H), 1250~1100 (C-H), 1664.15 (C=O), 1598.57 (N-H), 1487.87, 1466.65 (C-H), 949.64 (C-O), 763.71 (C-H).

13-2-13-2CH_3_CHOHCOO^−^: Mass Spectra: Cationic scanning *M/E*: 285.3; Anion scanning *M/E*: 88.9; FTIR Spectra (KBr, v, cm^-1^): 3394.04 (N-H), 3000~2800 (C-H), 1300~1150 (C-H), 1648.39 (C=O), 1593.47 (N-H), 1487.81, 1465.90 (C-H), 1125.17 (O-H), 947.71 (C-O), 686.18 (C-H).

15-2-15-2HCOO^−^: Mass Spectra: Cationic scanning *M/E*: 285.3; Anion scanning *M/E*: 45.0; FTIR Spectra (KBr, v, cm^-1^): 3300.56 (N-H), 3000~2800 (C-H), 1250~1100 (C-H), 1635.94 (C=O), 1597.42 (N-H), 1488.39, 1467.90 (C-H), 948.44 (C-O), 764.70 (C-H).

15-2-15-2CH_3_COO^−^: Mass Spectra: Cationic scanning *M/E*: 285.3; Anion scanning *M/E*: 59.0; FTIR Spectra (KBr, v, cm^-1^): 3351.01 (N-H), 3000~2800 (C-H), 1280~1140 (C-H), 1635.94 (C=O), 1595.02 (N-H), 1488.42, 1465.90 (C-H), 948.37 (C-O), 776.27 (C-H).

15-2-15-2CH_3_CHOHCOO^−^: Mass Spectra: Cationic scanning *M/E*: 285.3; Anion scanning *M/E*: 88.9; FTIR Spectra (KBr, v, cm^-1^): 3304.23 (N-H), 3000~2800 (C-H), 1300~1150 (C-H), 1640.17 (C=O), 1552.24 (N-H), 1467.24,1377.70 (C-H), 1124.55 (O-H), 964.06 (C-O), 760.83 (C-H).
