# Supplementary material for: The surface adsorption, aggregate structure and antibacterial activity of Gemini quaternary ammonium surfactants with carboxylic counterions
Source: R Soc Open Sci. 2019 Aug 28;6(8):190378. doi: 10.1098/rsos.190378 (PMC6731746; doi:10.1098/rsos.190378)
Supplement: Figure S1 [file rsos190378supp2.docx]

**
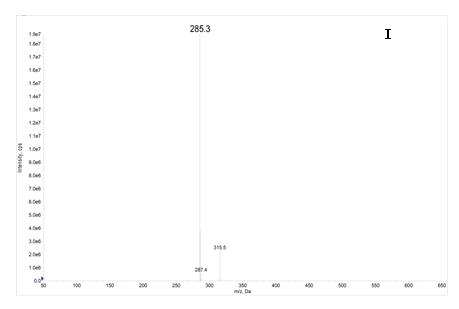
**


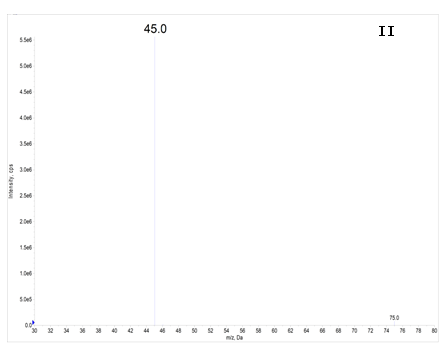


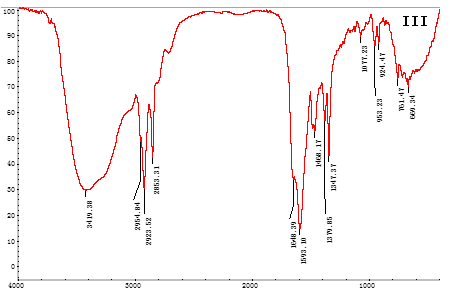


**Figure S1** Cationic scanning mass spectra (I), Anionic scanning mass spectra (II) and FTIR spectra (III) of 11-2-11-2HCOO^−^
