# Supplementary material for: The surface adsorption, aggregate structure and antibacterial activity of Gemini quaternary ammonium surfactants with carboxylic counterions
Source: R Soc Open Sci. 2019 Aug 28;6(8):190378. doi: 10.1098/rsos.190378 (PMC6731746; doi:10.1098/rsos.190378)
Supplement: Figure S2 [file rsos190378supp3.docx]

**Figure S2** Plots of conductivity (*κ*) against temperature of 11-2-11-2Y (A), 13-2-13-2Y (B), and 15-2-15-2Y (C), I: Y = HCOO^−^, II: Y = CH_3_COO^−^, and III: Y = CH_3_CHOHCOO^−^
