# Supplementary material for: The surface adsorption, aggregate structure and antibacterial activity of Gemini quaternary ammonium surfactants with carboxylic counterions
Source: R Soc Open Sci. 2019 Aug 28;6(8):190378. doi: 10.1098/rsos.190378 (PMC6731746; doi:10.1098/rsos.190378)
Supplement: Figure S4 [file rsos190378supp5.docx]

**Figure S4** Plots of 10^2^Λ vs *c*^0.5^ for 11-2-11-2CH_3_COO^−^ (I), 13-2-13-2CH_3_COO^−^ (I), 15-2-15-2HCOO^−^ (III), and 15-2-15-2CH_3_COO^−^ (IV) at 25 ^o^C
