# Supplementary material for: The surface adsorption, aggregate structure and antibacterial activity of Gemini quaternary ammonium surfactants with carboxylic counterions
Source: R Soc Open Sci. 2019 Aug 28;6(8):190378. doi: 10.1098/rsos.190378 (PMC6731746; doi:10.1098/rsos.190378)
Supplement: Figure S5 [file rsos190378supp6.docx]

**Figure S5** *R_h_* distribution of *n*-2-*n*-2Y, A: 2×CMC, B: 1×10^-3^mol·L^-1^, I: *n*=11, II: *n*=13, III: *n*=15, Y=HCOO^−^ (■), CH_3_COO^−^ (●), and CH_3_CHOHCOO^−^ (▲) in aqueous solution
